# The role of ENSO in understanding changes in Colombia's annual malaria burden by region, 1960–2006

**DOI:** 10.1186/1475-2875-8-6

**Published:** 2009-01-08

**Authors:** Gilma Mantilla, Hugo Oliveros, Anthony G Barnston

**Affiliations:** 1 International Research Institute for Climate and Society, Earth Institute at Columbia University, Lamont-Doherty Earth Obs., 61 Route 9W, Palisades, NY 10964, USA; 2 Econstat, Calle 75# 5-26 No. 701, Bogota, Colombia

## Abstract

**Background:**

Malaria remains a serious problem in Colombia. The number of malaria cases is governed by multiple climatic and non-climatic factors. Malaria control policies, and climate controls such as rainfall and temperature variations associated with the El Niño/Southern Oscillation (ENSO), have been associated with malaria case numbers. Using historical climate data and annual malaria case number data from 1960 to 2006, statistical models are developed to isolate the effects of climate in each of Colombia's five contrasting geographical regions.

**Methods:**

Because year to year climate variability associated with ENSO causes interannual variability in malaria case numbers, while changes in population and institutional control policy result in more gradual trends, the chosen predictors in the models are annual indices of the ENSO state (sea surface temperature [SST] in the tropical Pacific Ocean) and time reference indices keyed to two major malaria trends during the study period. Two models were used: a Poisson and a Negative Binomial regression model. Two ENSO indices, two time reference indices, and one dummy variable are chosen as candidate predictors. The analysis was conducted using the five geographical regions to match the similar aggregation used by the National Institute of Health for its official reports.

**Results:**

The Negative Binomial regression model is found better suited to the malaria cases in Colombia. Both the trend variables and the ENSO measures are significant predictors of malaria case numbers in Colombia as a whole, and in two of the five regions. A one degree Celsius change in SST (indicating a weak to moderate ENSO event) is seen to translate to an approximate 20% increase in malaria cases, holding other variables constant.

**Conclusion:**

Regional differentiation in the role of ENSO in understanding changes in Colombia's annual malaria burden during 1960–2006 was found, constituting a new approach to use ENSO as a significant predictor of the malaria cases in Colombia. These results naturally point to additional needed work: (1) refining the regional and seasonal dependence of climate on the ENSO state, and of malaria on the climate variables; (2) incorporating ENSO-related climate variability into dynamic malaria models.

## Background

Without doubt, epidemiologists, health management workers and health policy makers are very concerned about the potential impact that climate variability and climate change could have on human health. There is clear evidence that climate change is occurring in different parts of the world [1], and several hypotheses exist regarding the effects that those changes will have on the number of people suffering or dying from infectious diseases, heat waves, floods, storms, fires and droughts. Of equally great concern are the impacts of such "climate events" on the health care systems of the affected countries [2].

The impact of climate change on aspects of health has been actively discussed in the literature [3,4]. Experts suggest that global climate change could be the major driving mechanism behind increases in climate-sensitive diseases such as malaria. The World Health Organization (WHO) [5] suggested that an increase of about 6% in malaria incidence during 2000 in some middle-income countries could be attributed to climate change.

On a shorter time-scale, the Fourth Report of the Intergovernmental Panel on Climate Change (IPCC) concluded, using a systematic review of a number of studies, that the impacts of the El Niño/Southern Oscillation (ENSO) on the risk of malaria epidemics is well established in parts of Southern Asia and South America. ENSO is a cyclic phenomenon whose frequency is 2 to 7 years (i.e., irregular) and is the second strongest natural driver of climate variability, the first being normal seasonal variability. This oscillation has two different phases: a warm episode known as El Niño; and a cold episode called La Niña, where warm and cold refer to the direction of departure from average of the equatorial Pacific sea surface temperature (SST), a fundamental indicator of the ENSO state.

**Figure 1:**
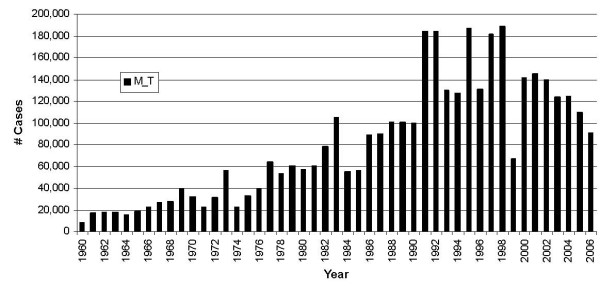
**Colombia's annual total number of malaria cases, 1960–2006**. Annual totals shown are for all types of malaria for all of Colombia. The El Niño years are 1965, 1972, 1982, 1983, 1987, 1991, 1992, 1997, and 2002; La Niña years are 1971, 1974, 1975, 1988 and 1999. The criterion for identifying these years is provided below, in the section describing the data.

Research has suggested a significant relationship between the state of ENSO and epidemic malaria in a number of tropical and subtropical regions, such as Kenya [6], Botswana [7], southern Africa more generally [8], and several countries in Asia and South America [9]. Such linkage between malaria and year-to-year-climate variability, coupled with some ability to predict ENSO-related, as well as some other climate variations [10,11], extend the lead time possible in organized malaria early warning systems over what can be done on the basis of real-time rainfall monitoring alone. Several studies of Colombia's climate variability and human health have been carried out on a national level. These have shown a significant positive association between the warm phase of ENSO and the number of cases of malaria [12-14]. However, malaria cases have noticeably diminished since 2000 (Figure 1). Thus, it seems natural to re-examine the question of the impact of ENSO on malaria in Colombia. Despite the valuable contributions of studies carried out to date for Colombia, it is necessary to extend and deepen the knowledge of the impact of climatic variability on Colombia's malaria transmission at the regional level.

As malaria case data has increased in temporal resolution beyond annual totals, it is becoming increasingly feasible to examine malaria's seasonal as well as regional dependence. Spatial variation of ENSO's effects on malaria within Colombia for given times of the year is of great importance, given the country's highly varied geography. Colombia provides a challenging test case in view of its geographical contrasts, annual cycle of rainfall and temperature, low endemic malaria condition, and recent downward trend in malaria cases.

### Malaria cases and climate, under improving institutional policies

Colombia is a tropical country located in northern South America with latitude range of 0°–11°N, longitude range 78°–66°W, and population approximately 44 million in 2007. In studying malaria in Colombia, several critical aspects need to be considered as drivers of the history of the malaria cases. Such influential factors need to be defined and studied to understand Colombia's previous, present and future performance of malaria control and surveillance.

Colombia has experienced three major institutional health system regimes during the last 50 years, and changes from one to another markedly affected its malaria control policy (Table 1). There was initially a Malaria Eradication Service (MES), an institutional program outside of the Ministry of Health. Then in 1974, a Direct Campaign began, that used the MES' framework but was operated directly by the Ministry of Health. Finally, since 1993 Colombia has functioned under a thoroughly reformed National Health Care System, including a dispersion of decision-making (decentralization) through which each state and/or municipality assumes responsibility to respond with control and intervention measures for any health issue, such as malaria. An important hypothesis to assess is that of a causal relationship between the increasing number of malaria cases seen through the mid-1990s, followed by the subsequent decline, and the changes in the malaria control policy and its institutional configuration [15].

**Table 1:** Colombia's Institutional framework of Malaria Control Programmes: 1960–2006^♦^

**Period**	**Health Care System**	**Malaria type of Intervention**	**Type of institutional Structure**	**Main Activities**
1960 – 1974	Unstructured Health System (ISS, Cajanal) -Public-	Malaria Eradication Service (MES^+^)	Vertical	DDT spraying, active case detection, treatment

1974 – 1993	Structured National Health System -Public-	Direct Campaign	Mix^♣^	Active and passive detection, treatment

1994–2006	General Social Security System on Health -Public, Private-	Vector Borne Disease Control (VBDC)	Horizontal^±^	For selective and regulated control intervention*

^♦ ^See also Valero, M., 2006, [16] for more detail about the institutional arrangement.

+ MES was independent from the Ministry of Health

^♣ ^It used MES' framework, but was operated directly by the Ministry of Health

^± ^It used a National Framework (VBDC) but it is operated by states and municipalities

* Early diagnosis, bed nets, insecticide spraying and treatment

The first framework prevailed until 1974, the second from 1974 to 1993, and the third from 1994 until present.

Colombia is a party to the United Nation Convention on Climate Change and has signed and ratified the Kyoto Protocol. The country has also developed legal and institutional frameworks for coordinating climate change issues. As a party of the Convention, Colombia produced in 2001 its First National Communication (NC-1) where the Colombian government identified malaria and dengue as the diseases of primary concern driven by changes in climate [16]. According to the Fourth Report of the IPCC, the global average surface temperature is likely to increase in the range 2° to 4.5°C during the 21^st ^century, with a best estimate of about 3°C [1]. This global warming will increase the likelihood of vector borne diseases. As a consequence, most tropical areas such as Colombia could expect to observe malaria incidence increases not only in their endemic-prone areas, but also in their more epidemic-prone highlands where human populations have low immunity rates to malaria parasites (Table 2).

**Table 2:** Colombia's 2005 Census Population. Profile by Threshold Elevation of Counties population

Threshold Elevation	Average Elevation* (masl)	2005 Census Population^+^		
		
		Total	Urban Area	Rural Area
Unknown Elevation	.	9,472	8,047	1,425
Between (0, 400] masl	146.6	11,331,631	7,835,910	3,495,721
Between (400, 1,000] masl	685.0	4,402,219	2,733,513	1,668,706
Between (1,000, 1,600] masl	1,278.5	7,466,660	5,551,976	1,914,684
Between (1,600, 2,000] masl	1,783.4	5,011,438	3,457,768	1,553,670
More than 2,000 masl	2,623.4	14,667,172	12,299,388	2,367,784

ALL	1,311.7	42,888,592	31,886,602	11,001,990

* Source: NOAA National Geophysical Data Center for Colombia's counties. "masl" denotes **m**eters **a**bove the **s**ea **l**evel.

^+ ^Source: DANE – Colombia's National Department of Statistics.

The elevation data comes from the NOAA National Geophysical Data Center, for Colombia's counties. The population data comes from 2005 census data from DANE (Colombia's National Department of Statistics).

### Colombia's malaria cases, 1960–2006

Malaria is caused by protozoan parasites (*Plasmodium*) that are transmitted by the bite of infected adult female *Anopheles mosquitoes*. Widespread in tropical and subtropical regions, the disease infects between 300 to 500 million people every year, and causes one to three million deaths annually, mostly in Saharan Africa. It is important to note that Colombia's malaria surveillance programs since 1960 have been based on blood tests and clinical follow-ups of positive cases. This is an important difference from many of the African countries where the predominant tool for malaria diagnosis is based on clinical symptoms, such as febrile illness, for treatment decisions. This symptom-based method usually results in over-diagnosis of malaria cases [17].

Malaria is still a major public health concern in Colombia, as shown in Figure 1. Nearly 85% of Colombia's rural territory below 1,600 meters above sea level (masl) has climatic, geographic and epidemiological characteristics suitable for malaria transmission. Based on the NOAA National Geophysical Data Center topographic elevation data for Colombia and Colombia's DANE county Census of 2005, 64.3% of Colombia's total rural population lives in such territory, and is at highest risk for malaria (Table 2).

During the past four decades, Colombia has had unstable/epidemic periods, each lasting from two to seven years. During the period 1960 to 1998, Colombia's malaria cases tended to increase, exceeding 187,000 cases by 1998. Although the number has remained above 100,000 during the last two decades, the trend has switched from positive to negative since the late 1990s. However, the problem is still important, not only because of the still high case numbers, but also because of the potential effects of global warming on the underprivileged populated suburban areas that have suitable geographical and climate conditions for malaria vectors. Those areas are inhabited by peasants and poor people who have "chosen" to move there because of systematic violence or lack of public goods and services in their previous rural areas, or by choice due to a perceived improvement in potential for a good existence.

**Figure 2:**
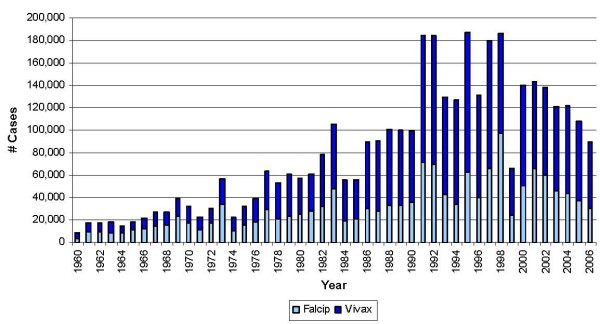
**Colombia's annual total number of malaria cases, 1960–2006, by type of parasite**. The proportion of the annual total cases identified as *Plasmodium falciparum *(light blue part of bar) and *Plasmodium vivax *(blue part of bar). *Plasmodium malariae *(not shown) makes up only approximately 1% of the total.

Several malaria vectors have been identified in Colombia, the most important of which are *Anopheles albimanus*, *Anopheles darling *and *Anopheles nunez – tovar *[18]. In the case of the parasites, *Plasmodium vivax*, *Plasmodium falciparum *and *Plasmodium malariae *have been identified. It is important to note a shift of the predominant type of parasite since 1974, in which malaria cases due *to P. vivax *have become more numerous than those due to *P. falciparum *(Figure 2), lately having an approximate proportion of 64%. Colombia's overall malaria mortality has significantly decreased and this trend continues. In the most recent decade, mortality has been approximately 130 to 150 deaths per year [19].

There is great geographic and cultural diversity in Colombia. Factors such as climatic variation, and violence related to socio-economic polarity, have intensified in recent years. The presence of displaced populations due to armed conflict and poverty in rural areas motivates us to identify, understand and predict the distinct epidemiological stages of Colombia's malaria transmission, especially the early stages when mitigation measures are possible. The problem is particularly difficult, given the complex interactions between ecological and social processes occurring in disease-prone areas in the context of changing climatic conditions. These background conditions in the disease ecology of malaria pose unique challenges for malaria surveillance, treatment and prevention in Colombia.

**Figure 3:**
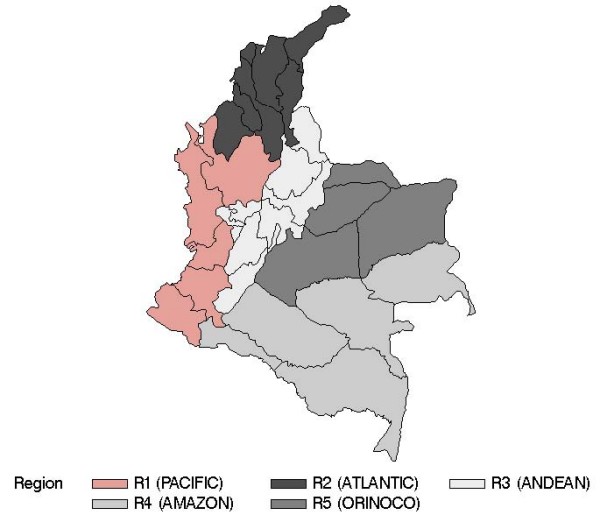
**Colombia's malaria regions**. Colombia's five malaria regions match the five geographical and climatological homogeneous regions (Table 3): R1 (Pacific); R2 (Atlantic); R3 (Andean); R4 (Amazon); and R5 (Orinoco).

**Figure 4:**
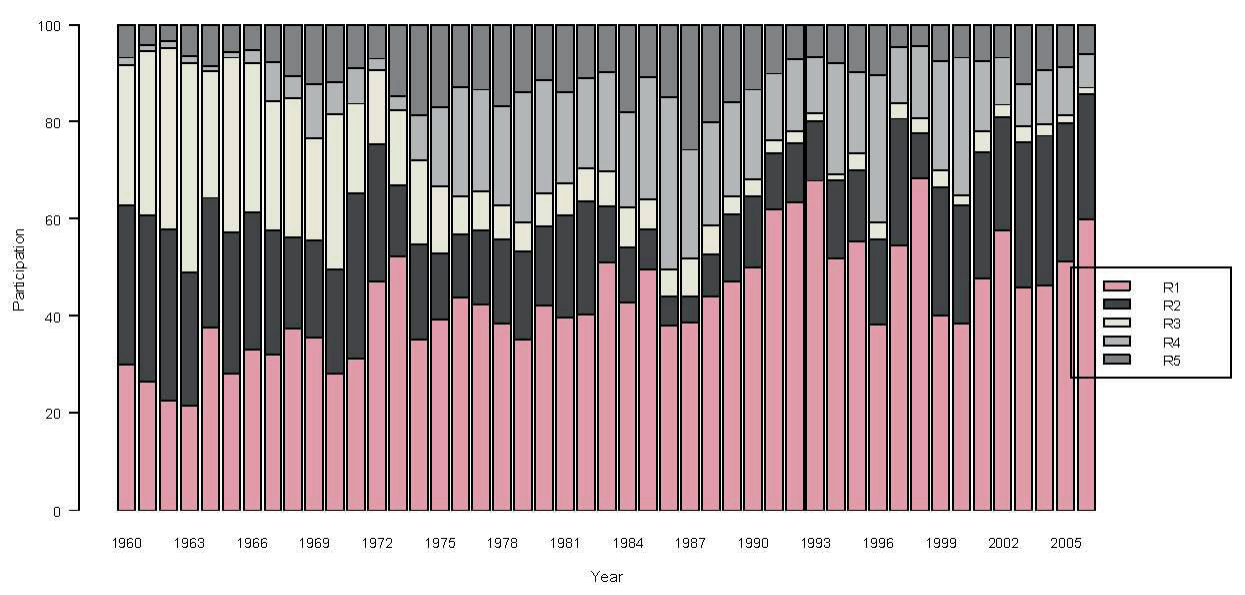
**Regional contributions to Colombia's total malaria cases, 1961–2006**. Bar colors show annual regional proportion of contribution to the total of the malaria cases.

**Figure 5:**
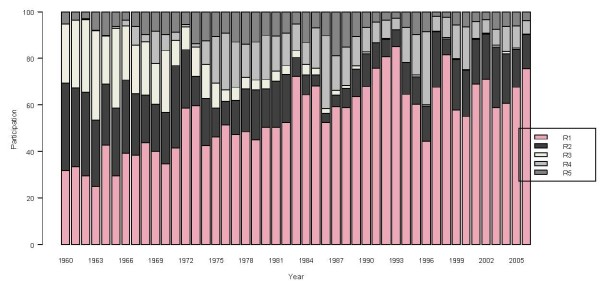
**Regional contributions to Colombia's *Falciparum *malaria cases, 1961–2006**. Bar colors show annual regional proportion of contribution to the total of the *Falciparum *malaria cases.

**Figure 6:**
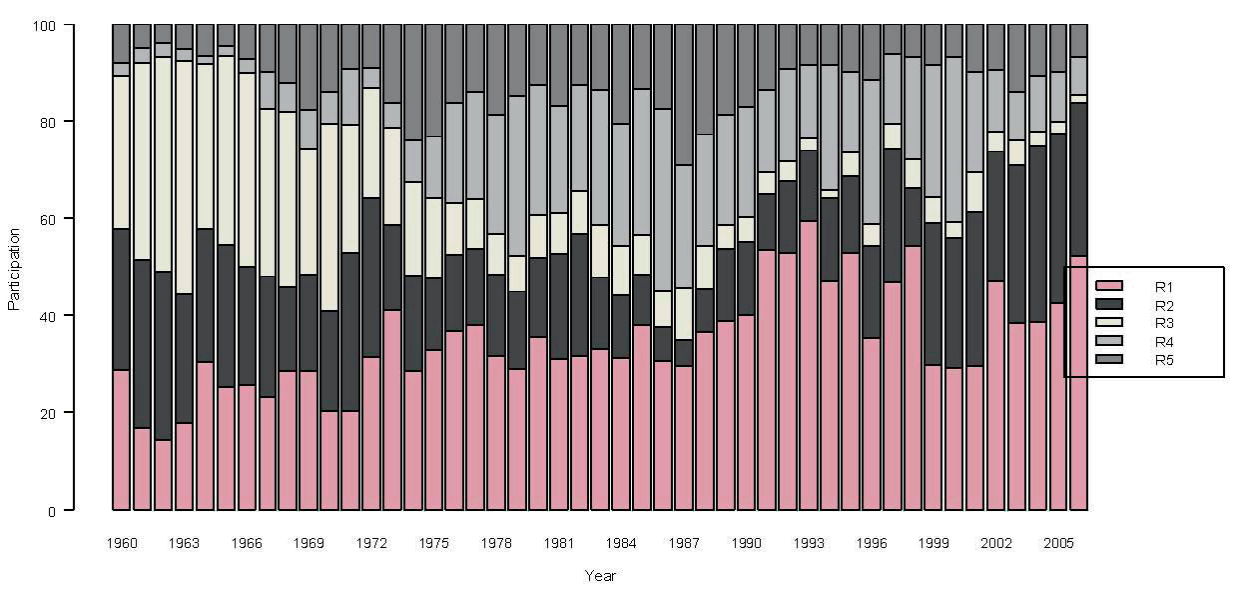
**Regional contributions to Colombia's *Vivax *malaria cases, 1961–2006**. Bar colors show annual regional proportion of contribution to the total of the *Vivax *malaria cases.

Following its geographical contrast, Colombia is often divided into five regions: Pacific (R1), Atlantic (R2), Andean (R3), Amazon (R4) and Orinoco (R5) (Table 3, Figure 3) [20]. The evolution of malaria cases differs across these regions, as shown by the time series of the relative regional proportions of malaria cases from 1960 to 2006 (Figure 4). Malaria cases are seen to be most concentrated in the Pacific region. A major re-emergence is noted in the Atlantic region since 1986, in contrast with the negligible level in the Andean region by the end of 2006. The two predominant *Plasmodium *types, *falciparum *and *vivax*, together represent almost 99% of Colombia's malaria cases. The historical proportions of the regional shares of these two types are presented in Figures 5 and 6, respectively. An increasingly predominant pattern is noted in the Pacific region for *P. falciparum *(Figure 5). The regional share of *P. vivax*, as might be expected, has a pattern more similar to that noted for the total malaria cases (Figure 3).

**Table 3:** Regional definition of Colombia's States^♣^

**Region**	**Name**	**States**
R1	Pacific	Nariño, Cauca, Valle del Cauca, Choco, Antioquia
R2	Atlantic	Atlantico, Bolivar, Cesar, Cordoba, Guajira, Magdalena, Sucre
R3	Andean	Cundinamarca, Boyaca, Caldas, Huila, Norte de Santander, Quindio, Risaralda, Santander, Tolima
R4	Amazon	Caqueta, Putumayo, Amazonas, Vaupes, Guaviare, Guainia
R5	Orinoco	Meta, Arauca, Casanare, Vichada

^♣ ^This classification is similar to one used by Gagnon et al (2002), [20]

### Data

This section describes the origin and content of the available data and the methodology designs employed to analyse malaria cases and climate factors. Colombia's malaria cases data comes from the national s infectious disease information system, called SIVIGILA. Malaria cases were recorded at the state level from 1960 to 2000 on a yearly basis. Then, from 2001 to 2006, the data was registered at the more highly resolved county level, and on a weekly basis. The malaria cases data are confirmed cases of reports submitted by State's Secretary of Health to the Ministry of Health from 1960 to 2000 and to the National Health Institute (INS), from 2001 to 2006. For the regional analyses the data were aggregated using the regions defined in Table 3.

The ENSO data comes from the Oceanic Niño Index (ONI) calculated by the U.S. National Oceanic and Atmospheric Administration (NOAA) using version 3 of the Extended Reconstructed Sea Surface Temperature (ERSST.v3) dataset. The classification of ENSO episodes as Warm (El Niño) or Cold (La Niña) is based on a threshold of ± 0.5°C for the ONI for a 3 month running mean of SST anomalies in the Niño 3.4 region in the east-central equatorial Pacific (5°N – 5°S, 120–170°W) [21], derived from the 1971–2000 base period. NOAA's ENSO data set contains monthly observations of the Niño 3.4 ENSO-related SST index from January 1950 to within three months of the present month.

**Figure 7:**
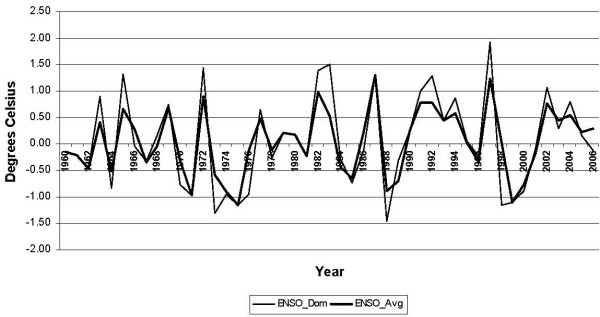
**Annual measure of ENSO status, 1960–2006**. The index of ENSO is the sea surface temperature (SST) averaged by month over the east-central tropical Pacific region called "Niño 3.4" (5°N – 5°S, 120–170°W) [21]. The thick line represents ENSO_Avg, which is simply the average of the SST anomaly (°C) in the region over the 12 months of the year. The thin line represents ENSO_Dom, which is the average SST during only the months having the ENSO state (La Niña, neutral, or El Niño) that existed for the greatest number of consecutive months during the given year. A first-order categorization of the ENSO status of each year, listed in the caption of Fig.1, is based on whether the weighted average of 1/3 × (ENSO_Dom) + 2/3 × (ENSO_Avg), is above 0.85°C (El Niño), or below -0.85°C (La Niña).

The monthly Niño 3.4 SST data was used to construct two ENSO variables for each year, each defining a particular aspect of the ENSO state. First, the monthly ENSO data were averaged for the year, and denote this as ENSO_Avg. Secondly, only those consecutive monthly values representing the most frequently occurring, or predominant, ENSO state were averaged for the given year. This is defined as the largest number of consecutive months, greater than or equal to five months, being El Niño, La Niña, or neutral. In the case of a tie between an El Niño (or La Niña) and a neutral episode, the year is categorized as the non-neutral ENSO state. This variable is denoted as ENSO_Dom (for ENSO_dominant). The evolution of the two ENSO indicators is shown in Figure 7. As one might expect, ENSO_Dom has higher amplitude of variability than ENSO_Avg. A weighted average of ENSO_Avg and ENSO_Dom (with ENSO_Avg weighted double) is used to determine the ENSO status of each year, using ± 0.85°C as cutoffs, listed in the caption of Fig. 1. (The seasonal cycle of ENSO does not mesh very well with the calendar year, as it typically straddles two calendar years). Table 4 shows the dependent and independent variables used in the models.

**Table 4:** Colombia's malaria models: predictand and predictor variable definitions

**Predictand Variable**	**Description**
M_T	Colombia's Total Malaria Cases
M_R1	Colombia's Total Malaria Cases in West (Pacific) Region
M_R2	Colombia's Total Malaria Cases in North (Atlantic) Region
M_R3	Colombia's Total Malaria Cases in Midwest (Andean) Region
M_R4	Colombia's Total Malaria Cases in Southeast (Amazon) Region
M_R5	Colombia's Total Malaria Cases in Northeast (Orinoco) Region

**Predictor Variable**	**Description**

ENSO_Avg	Yearly index based on average of the 12 monthly data for each year (°C)
ENSO_Dom	Yearly index based on average of only those consecutive months having the dominant ENSO state (La Niña, neutral, or El Niño) (°C)
Trend1	Time reference variable t = 1, 2,..., 47 associated with 1960–2006 period
Trend2	Time reference variable t = 1, 2, 3...8 associated with 1999 – 2006 period; 0 otherwise (1960–1998)
**Vextre♣**	Dummy variable: takes value of 1 in 1999; 0 otherwise

^♣ ^This dummy variable is associated with the significant drop of malaria cases in 1999. It can be attributed to a change in the registration process of the infectious diseases surveillance system during the embedding period. The observation in 1999 has a high high leverage in any regression model, and thus it is treated as an outlier to diminish its impact on the estimation process.

The predictand (malaria case numbers) are for Colombia as a whole, and for each of the 5 regions defined in Table 3 and Figure 3. The predictors include two indices of the ENSO state, two trends, and the vextre for special treatment for the year 1999.

## Methods

The literature about the association between malaria and ENSO or climate variability is fairly large and has a wide variety of flavors, including the nature and level of sophistication of the methodological approach. Because the number of malaria cases (the dependent variable) must be a positive integer, one needs to consider a set of probability distributions suitable for this type of random variable. The methods used for such analyses vary from: (i) correlation analysis for the number of cases (or relative proportion of malaria cases – incidence) versus ENSO measures, which varies by geographical region, by the portion of the overall study period, and by the level of aggregation of the malaria cases (see [13-15] for Colombia, for instance); (ii) contingency table analysis that use various thresholds defining malaria rate and ENSO categories [20]; (iii) regression analysis using either malaria cases or relative changes (delta log method) and a set of independent (predictor) variables such as ENSO, or climate variables influenced by ENSO such as precipitation and temperature [22,23]; and even (iv) state space models that deal with, for instance, dynamics problems that can arise when working with count data [24].

In the case of regression analysis (iii above) it is important to recognize whether the dependent variable takes on integer values (e.g. number of cases), or real values (e.g. incidence index, or delta log transformations), since the model estimation procedure is driven by the scale definition of the dependent variable. For instance, for the former case, Poisson regression models, or negative binomial models, are more suitable than linear models based on Gaussian errors, which apply perfectly to the latest case [22,23].

Models for count data and time series models are highly sensitive to the manner in which the seasonal cycle [23] and trend patterns are handled – e.g. either removed from the data or allowed to remain in the data but treated in some way (e.g. stochastic trends, or stationary conditions around a trend) in the model design using variables or filters that can take account of such issues throughout the analysis. Here the latter approach was used, in which trend variables are broadly and generally included, in assessing the effects of ENSO on Colombia's total and regional malaria cases.

There is a tradeoff between the ways that seasonality and trend are treated. From a statistical estimation perspective, some degrees of freedom are lost if the trend component of the dependent variable is modeled, but some substantial drawbacks are avoided in doing so. For instance, when a moving average is used to estimate a smoothed trend line to be removed (before the modeling process begins), the length of the moving average is limited by the length of cycle of the dependent variable. Thus, a moving average filter becomes *ad-hoc *in this context if the length of the cycle is unknown. However, when trend variables are included in the model, their importance is formally tested in the model (with fewer degrees of freedom). An additional drawback in using a moving average is that the number of observations available to the model is diminished up to the length of the moving average, unless a taper is used at both ends.

During the 1960–2006 period, malaria cases showed two different trend patterns (Figure 1): a rising trend from 1960 to 1998, a sudden decline of cases in 1999, a return to a more normal level in 2000, and finally a more systematic decrease in cases between 2000 and 2006. Thus, two trend variables can be used to capture the trends, and a dummy variable (called "vextre") can capture the total pattern of the trends during this period (see Table 4 for detail).

Table 5 describes the general models considered in this study and the way the variables are used to estimate the model parameters. Poisson regression models (PRM), and Negative Binomial regression models (NBRM) are the baseline models used to test the influence of ENSO on Colombia's malaria cases. A Negative Binomial Distribution (NBD) nests the Poisson distribution under parameter restrictions (i.e., when *α *= 0; see Table 5). Thus, a model under NBD allows us to handle over dispersion or under dispersion conditions in a proper way. Such conditions are very common in studies such as this, and impossible to handle under a Poisson Distribution, given that its conditional mean and variance are equal, and an equidispersion condition is assumed (Table 5).

**Table 5:** Discrete probability distribution used to model Colombia's malaria cases

**Poisson Model** **(PM)**	**Negative Binomial Regression Model (NBRM)**	**Link Function**
*E*(*Y*_*t*_/*X*_*t*_) = *λ*_*t *_= exp(*X*_*t*_*β*) > 0	*E*(*Y*_*t*_/*X*_*t*_) = *λ*_*t *_= exp(*X*_*t*_*β*) > 0	Log
*V*(*Y*_*t*_/*X*_*t*_) = *λ*_*t *_= exp(*X*_*t*_*β*) > 0	*V*(*Y*_*t*_/*X*_*t*_) = *λ*_*t *_+ *α *(*λ*_*t*_)^2-*k*^; *k *= 0,1	Log

		

*E*(*Y*_*t*_/*X*_*t*_): Expected value of number of malaria cases in year t given the information of *X*_*t*_.		
*V*(*Y*_*t*_/*X*_*t*_): Variance of the number of malaria cases in year t given the information of *X*_*t*_.		
*β*: unknown set of parameters		
*α*: dispersion parameter [*α *> 0 over-dispersion; *α *< 0 under-dispersion]		
*Y*_*t*_: dependent variable: Number of Malaria cases per year (Total, R1,..., R5)		
*X*_*t*_: set of independent or explanatory variables.		
*Y*_*t*_: {Mal_Tot, Mal_R1,..., Mal_R5}: set of dependent variables.		
*X*_*t*_: {Base Line Trend, ENSO Measure} = {BLT, ENSO}		
BLT: {Intercept, Trend1, Trend2, Vextre}: some or all of them		
ENSO: {ENSO_Avg, ENSO_Dom}: one of them		

**Probability Distribution (PD)**		

Poisson PD: $P\left[Y_{t}=y_{t}\right]=\frac{{\left(\lambda_{t}\right)}^{y_{t}}\exp(\lambda_{t})}{y_{t}!}$		

Negative Binomial PD: $P\left[Y_{t}=y_{t}\right]=\frac{\Gamma \left(y_{t}+\frac{1}{\alpha }\right)}{\Gamma \left(y_{t}+1\right)}\frac{1}{\Gamma \left(1+\alpha \right)}\frac{{\left(\alpha \lambda_{t}\right)}^{y_{t}}}{{\left(1+\alpha \lambda_{t}\right)}^{y_{t}+\frac{1}{\alpha }}}$		

The expected value and variance of the Poisson Regression Model and the Negative Binomial Regression Model are shown at top, their distributions are defined at bottom, and symbols are defined in middle.

## Results

Results of the estimation of the model parameters (Table 5) and performance are presented first for the PRM and NBRM models for Colombia's total malaria cases (Table 6), and then for the individual regions as defined above in Table 3 (as well as in Additional Files 1 and 2). In all cases, both of the potential measures of ENSO (ENSO_ Avg and ENSO_Dom; Figure 7) are considered to determine if one or both helps to explain the behaviour of malaria cases. The results for Colombia's total malaria cases (Table 6) suggest the following:

**Table 6:** Results of Poisson Regression Model: PRM; Negative Binomial Regression Model: NBRM.

**Dependent Variable:**	**Mal_Tot**				**Mal_Tot**			
**Type of Model**	**PRM**		**NBRM**		**PRM**		**NBRM**	

Nobs	47		47		47		47	

DF	42		42		42		42	

								

**ENSO Measure**	**ENSO_Avg**				**ENSO_Dom**			

								

**Model**								

Deviance	114465.6		47.3		119277.8		47.4	

Deviance/DF	2725.37		1.13		2839.95		1.13	

								

**Parameters**	**PRM**	**S**	**NBRM**	**S**	**PRM**	**S**	**NBRM**	**S**

Intercept	9.6067	***	9.5399	***	9.5926	***	9.5293	***

Trend1	0.0650	***	0.0677	***	0.0657	***	0.0683	***

Trend2	-0.1545	***	-0.1640	***	-0.1541	***	-0.1625	***

Vextre	-0.7768	***	-0.7941	***	-0.8539	***	-0.8901	***

ENSO Measure	0.1512	***	0.1626	**	0.0937	**	0.0893	**

Dispersion♣			0.0386				0.0414	

**Tests**								

W:BLT vs (BLT + ENSO ]	29602.6	***	9.2	***	24790.4	***	5.9	**

Colombia's Total Malaria Cases Models: Mal_Tot. Yearly data 1960–2006.

Nobs: Number of observations available for the model;

DF: Degrees of Freedom; BLT: Some or all of Base Line Trends (including vextre)

Test: Wald Test, W, LR; High value or number of stars means reject in favor of the last model used in the test, [];

S: Significance: P-value ≤ 0.01: ***; 0.01 < P-value ≤0.05: **; 0.05 ≤ P-value < 0.10: *; P-value > 0.1 (NS)

♣: All confidence intervals at 95% confidence of the dispersion parameter do not include zero inside their boundaries

Coefficients and their approximate statistical significances are shown for both Poisson (PRM) and Negative Regression Binomial (NBRM) models, each for either of the two ENSO indices (ENSO_Avg and ENSO_Dom).

• The NBRM is the more suitable model for the behaviour of the total malaria cases in Colombia. The Deviance/DF measure is approximately unity when the model fit to the data is favorable, even with some dispersion.

• The trend variables and ENSO measures are significant. In all cases the inclusion of the ENSO variable in the model maximizes the likelihood function (Wald test), and the values of the parameters are the expected ones, given the particular behaviour of Colombia's total malaria cases described above.

• Since a log-link function was used to estimate the model parameters, one can say that a change of 1° C on ENSO_Avg or ENSO_Dom measures changes the expected value of Colombia's total malaria cases by 17.7% or 9.3%, respectively.

The results differ among the five individual regions defined in Table 3 and Figure 3. Additional Files 1 and 2 show results for each region when ENSO is represented by ENSO_Avg and ENSO_Dom, respectively. The regional results can be highlighted as follows:

• For all five regions, the NBRM statistics suggest a better fit than the PRM for the malaria cases. In similar fashion to the results for total malaria cases, the Deviance/DF is close to 1.0, indicating a favorable model fit to the data. That a near-unity result indicates a favorable model goodness-of-fit is based on the fact that deviance is distributed as *χ*^2 ^(DF) with an expected value equal to the number of degrees of freedom, DF.

• For the Pacific region 1 (R1) and the Atlantic Region (R2), the models show that malaria cases are positively and significantly associated with ENSO behaviour (i.e. high during El Niño), for both ENSO_Avg and ENSO_Dom variables.

• For Andean, Amazon and Orinoco regions, R3, R4 and R5 respectively, neither ENSO_Avg nor ENSO_Dom shows a significant relationship with malaria cases during this period.

• Since a log-link function was used to estimate the models, one can say that a change of 1° C on ENSO_Avg or ENSO_Dom measures will change the expected value of malaria cases in Region 1 by 22.9% and 9.6%, respectively. For Region 2 these figures are 23.4% and 19.4%, respectively.

## Discussion

Colombia's malaria situation has changed during the last four decades from an incipient recognizable problem at the beginning of the 1960s, to a serious and complex problem with a systematically upward trend of cases until 1998. Since the late 1990s, malaria cases have slowly but steadily diminished through 2006. During this period Colombia's the predominant *Plasmodium *malaria cases shifted from falciparum to vivaxwith some regional differences as presented (Figures 4, 5, 6). The prevalence of *P. falciparum *in the Pacific region and to a lesser degree in the Atlantic region, compared to a relatively greater proportion of *P. vivax *in the other regions, is a general feature one must consider in designing malaria health interventions such as early warning systems for the country and its regions.

When models include trend variables to capture the relatively slowly changing features of malaria cases in Colombia, the influence of ENSO on malaria becomes discernible using even the rough measures of ENSO defined here. An ENSO influence helps to explain the behaviour of Colombia's malaria cases both at the national level and for some individual regions, such as the Pacific and the Atlantic regions – those two regions accounting for 60% to 75% of Colombia's malaria cases. From the health management and policy makers' perspective, it is very useful to have a regional differentiation of how ENSO changes could increase/decrease the number of malaria cases. This information will be relevant to allocate resources effectively in those regions.

The influence of ENSO-related climate anomalies on malaria cases should ideally be analysed by individual season, making possible a better defined series of events explainable in physical terms. While the malaria data is limited to annual resolution, monthly resolution exists for ENSO and Colombia climate data. The mean seasonal cycle of rainfall in some parts of the country is fairly uniform, but in other regions features a more clearly defined rainy season, or two rainy seasons. The ENSO cycle itself has preferred seasons of development, maturation and demise (April-June, November-January, and February-April, respectively). The lack of seasonal resolution in the malaria data does not preclude seasonally refined analyses of the effects of ENSO on rainfall in each region of Colombia. This would provide information about how ENSO influences malaria at each time of the year, including time lags between the ENSO state, the climate response and the consequent malaria response.

Ultimately, probabilistic forecasts of the ENSO state could be used to form probability forecasts of temperature and precipitation, which in turn could lead to predicted malaria levels, together with their uncertainties, throughout Colombia. In the case of some regions there may be a 1-year offset between the time when the ENSO state is most critical, and the most likely time of the corresponding malaria effects. Such instances are not captured in the current analysis design with unlagged annual temporal units. Also hidden in the current design are possible cases in which the ENSO phase reverses from the first one-third of the year to the second two-thirds (e.g. 1983, 1998). Seasonal refinement in the design of the climatic components of climate-malaria relationships is therefore one aspect of our future research plan. The existence of weekly resolved malaria data by municipality since 2001 in Colombia should be very useful for such seasonally differentiated studies, and utility will increase as the period of record increases.

Because the ENSO state is one of the most important known bases for determining the climate anomaly in Colombia [24], an ability to predict ENSO itself is necessary. ENSO predictability one to two seasons in advance is known to be moderately good for forecasts made between July and January, but only fair between February and May during the so-called "northern hemisphere ENSO predictability barrier". Prospects for further gradual improvement in ENSO prediction appear favorable [25]. Malaria prediction using seasonal climate forecasts may, therefore, be considered feasible, and may be expected to improve slowly over time, given the connections along the chain from predicting ENSO, then Colombia's upcoming seasonal climate, and finally effects of climate on malaria activity.

A similar strategy to predict malaria and provide early warning has been studied for Botswana [7]. For Botswana the malaria-determining rainy season occurs from November to March, a time of year for which ENSO is well predicted and in a mature phase, and a relationship between ENSO and rainfall anomaly is documented for that portion of southern Africa [24,26]. The season in which malaria occurs in Botswana is limited to approximately February to May, lagging the rainy season by two to three months. In Colombia the rainy season(s) tend to be more pervasive throughout the year, and effects of ENSO on climate, and of climate on malaria, need to be carefully seasonally differentiated to develop an effective malaria early warning system.

## Conclusion

Unlike previous studies of the relationship between Colombia's malaria cases and ENSO (or ENSO-associated climate responses), this study used a regional approach and sought to bridge the gap between recent studies that have updated Colombia's malaria data, the recent need for methods of dealing with trend issues, and the long-standing characteristic of malaria case data being count (integer) data. This study demonstrates that ENSO-related climate variability matters despite the presence of multiple background trends.

The existence of regional patterns and trends opens new areas of research. However, it is recognized that still greater effort will be necessary to introduce a dynamic model [27,28], and to use ENSO-related climate variables such as precipitation and temperature [29], as well as socio-economic indicators to understand the behaviour of Colombia's malaria. The analysis may need to be stratified by season to account for the seasonal dependence in the relationships of climate to ENSO, as well as the relationships of climate anomalies to malaria. Climate driven spatial patterns need to be included or derived according to the malaria cases behaviour using either multivariate analysis techniques or model-based indicators to develop individual risk indicators and total risk maps.

On the prediction side, one aspect that has to be acknowledged nowadays is the existence of Colombia's high frequency Malaria records – coming from the National Health Surveillance System – as well as monthly climate and ENSO anomaly data. Thus, the chances to improve malaria prediction models following the idea that the conditional distribution of malaria is a function of a set of independent variables plus a set of autoregressive and moving average terms will be very helpful. A promising experience in that context is found, for instance, for a Negative Binomial GARMA model which was used to explain the behaviour of poliomyelitis monthly cases count data [30].

## Competing interests

The authors declare that they have no competing interests.

## Authors' contributions

GM provided the malaria case data, offered expert experience and knowledge of the dynamics of the two major strains of malaria affecting Colombia's human population, as well as the evolution of Colombia's three main malaria control policies from 1960 to the present. She incorporated most of the malaria journal articles into formation of the purpose and motivation for this paper and assisted in developing the paper.

HO conceived and carried out the statistical analyses aimed at isolating and quantifying the influences of the changing malaria control policies and the ENSO-related climate variations on the malaria case numbers from 1960 to 2006 in Colombia as a whole, and in its geographical/climatic regions. He made the greatest contribution to composing the initial draft.

AGB provided knowledge of the seasonally and regionally dependent climate response to ENSO in Colombia, provided ENSO-related Pacific sea surface temperature data, and helped decide upon ENSO indices of relevance to malaria in Colombia. He critically reviewed and edited the manuscript for content, clarity and organization.

## Supplementary Material

Additional file 1 **Results of two statistical models relating malaria cases to ENSO, using ENSO_Avg**. Regression coefficients and their approximate statistical significances for Poisson and the negative binomial regression models, relating malaria cases to ENSO, accounting for base line trends and using ENSO_Avg to represent the annual ENSO state. Click here for file

Additional file 2 **Results of two statistical models relating malaria cases to ENSO, using ENSO_Dom.** Regression coefficients and their approximate statistical significances for Poisson and the negative binomial regression models, relating malaria cases to ENSO, accounting for base line trends and using ENSO_Dom to represent the annual ENSO state. Click here for file
