# Supplementary material for: The role of ENSO in understanding changes in Colombia's annual malaria burden by region, 1960–2006
Source: Malar J. 2009 Jan 8;8:6. doi: 10.1186/1475-2875-8-6 (PMC2661091; doi:10.1186/1475-2875-8-6)
Supplement: Additional file 1 — Results of two statistical models relating malaria cases to ENSO, using ENSO_Avg. Regression coefficients and their approximate statistical significances for Poisson and the negative binomial regression models, relating malaria cases to ENSO, accounting for base line trends and using ENSO_Avg to represent the annual ENSO state. [file 1475-2875-8-6-S1.doc]

**Additional File 1**

**Results of Poisson Regression Model: PRM ; Negative Binomial Regression Model: NBRM**

**Colombia’s Regional Malaria Cases Models : {Mal_R1,…,Mal_R5} vs {BLT + ENSO_Avg}.** Yearly data, 1960-2006

| **Dependent Variable** | **Mal_R1** | | |  | **Mal_R2** | | |  | **Mal_R3** | | |  | **Mal_R4** | | |  | **Mal_R5** | | |  |
| --- | --- | --- | --- | --- | --- | --- | --- | --- | --- | --- | --- | --- | --- | --- | --- | --- | --- | --- | --- | --- |
| **Type of Model** | **PRM** |  | **NBRM** |  | **PRM** |  | **NBRM** |  | **PRM** |  | **NBRM** |  | **PRM** |  | **NBRM** |  | **PRM** |  | **NBRM** |  |
|  |  |  |  |  |  |  |  |  |  |  |  |  |  |  |  |  |  |  |  |  |
| Nobs | 47 |  | 47 |  | 47 |  | 47 |  | 47 |  | 47 |  | 47 |  | 47 |  | 47 |  | 47 |  |
| DF | 42 |  | 42 |  | 44 |  | 44 |  | 44 |  | 44 |  | 42 |  | 42 |  | 43 |  | 43 |  |
|  |  |  |  |  |  |  |  |  |  |  |  |  |  |  |  |  |  |  |  |  |
| **ENSO Measure** | **ENSO_Avg** | | | | | | | | | | | | | | | | | | | |
|  |  |  |  |  |  |  |  |  |  |  |  |  |  |  |  |  |  |  |  |  |
| Deviance | 139916.3 |  | 47.6 |  | 71654.9 |  | 47.9 |  | 29680.7 |  | 48.1 |  | 102911.6 |  | 50.1 |  | 103439.6 |  | 49.5 |  |
| Deviance/DF | 3331.34 |  | 1.13 |  | 1628.52 |  | 1.09 |  | 674.56 |  | 1.09 |  | 2450.28 |  | 1.19 |  | 2405.57 |  | 1.15 |  |
|  |  |  |  |  |  |  |  |  |  |  |  |  |  |  |  |  |  |  |  |  |
| **Parameters** | **PRM** | **S** | **NBRM** | **S** | **PRM** | **S** | **NBRM** | **S** | **PRM** | **S** | **NBRM** | **S** | **PRM** | **S** | **NBRM** | **S** | **PRM** | **S** | **NBRM** | **S** |
| Intercept | 8.4232 | *** | 8.2856 | *** | 8.1880 | ******* | 8.2730 | ******* | 8.8313 | ******* | 8.8459 | ******* | 7.3003 | *** | 6.1392 | ******* | 8.0809 | *** | 7.4883 | ******* |
| Trend1 | 0.0805 | *** | 0.0859 | *** | 0.0485 | ******* | 0.0455 | ******* | -0.0148 | ******* | -0.0154 | ******* | 0.0859 | *** | 0.1314 | ******* | 0.0346 | *** | 0.0569 | ******* |
| Trend2 | -0.1928 | *** | -0.2059 | *** | NA |  | NA |  | NA |  | NA |  | -0.2977 | *** | -0.4556 | ******* | NA |  | NA |  |
| Vextre | -0.9927 | *** | -1.0932 | *** | NA |  | NA |  | NA |  | NA |  | -0.8870 | *** | -1.2171 | ***** | -0.8973 | *** | -1.2296 | ******* |
| ENSO | 0.2372 | *** | 0.2063 | *** | 0.2109 | ******* | 0.2104 | ****** | 0.1278 | ******* | 0.1178 | **NS** | -0.0602 | *** | 0.0938 | **NS** | 0.0478 | NS | 0.0162 | **NS** |
| Dispersion |  |  | 0.0822 |  |  |  | 0.1140 |  |  |  | 0.1415 |  |  |  | 0.4003 |  |  |  | 0.3174 |  |
| **Test** |  |  |  |  |  |  |  |  |  |  |  |  |  |  |  |  |  |  |  |  |
| W: BLT vs  [ BLT + ENSO] | 35868.3 | *** | 7.0 | *** | 11069.3 | ******* | 6.2 | ****** | 1349.8 | ******* | 1.7 | **NS** | 805.6 | *** | 0.3 | **NS** | 282.1 |  | 0.0 | **NS** |
|  |  |  |  |  |  |  |  |  |  |  |  |  |  |  |  |  |  |  |  |  |
| Nobs: Number of observations available for the model;  DF: Degrees of Freedom;  Test: Wald Test, W ; High value or number of stars means reject in favor of the last model used in the test, [ ];  S: Significance: P-value ≤ 0.01: *** ; 0.01 < P-value ≤ 0.05 : ** ; 0.05 ≤ P-value < 0.10 : * ; P-value >0.1 (NS)  : All confidence intervals at 95% confidence of the dispersion parameter do not include zero inside their boundaries  NA: Not available | | | | | | | | | | | | | | | | | | | | |

Coefficients and their approximate statistical significances are shown for both Poisson (PRM) and Negative Binomial Regression models (NBRM), using ENSO_Avg to represent the annual ENSO state.
