# Supplementary material for: The role of ENSO in understanding changes in Colombia's annual malaria burden by region, 1960–2006
Source: Malar J. 2009 Jan 8;8:6. doi: 10.1186/1475-2875-8-6 (PMC2661091; doi:10.1186/1475-2875-8-6)
Supplement: Additional file 2 — Results of two statistical models relating malaria cases to ENSO, using ENSO_Dom. Regression coefficients and their approximate statistical significances for Poisson and the negative binomial regression models, relating malaria cases to ENSO, accounting for base line trends and using ENSO_Dom to represent the annual ENSO state. [file 1475-2875-8-6-S2.doc]

**Additional File 2**

**Results of Poisson Model: PRM ; Negative Binomial Regression Model: NBRM**

**Colombia’s Regional Malaria Cases Models : {Mal_R1,…,Mal_R5} vs {BLT + ENSO_dom}.** Yearly data, 1960-2006

| **Dependent Variable** | **Mal_R1** | | |  | **Mal_R2** | | |  | **Mal_R3** | | |  | **Mal_R4** | | |  | **Mal_R5** | | |  |
| --- | --- | --- | --- | --- | --- | --- | --- | --- | --- | --- | --- | --- | --- | --- | --- | --- | --- | --- | --- | --- |
| **Type of Model** | **PRM** |  | **NBRM** |  | **PRM** |  | **NBRM** | **S** | **PRM** |  | **NBRM** |  | **PRM** |  | **NBRM** |  | **PRM** |  | **NBRM** | **S** |
|  |  |  |  |  |  |  |  |  |  |  |  |  |  |  |  |  |  |  |  |  |
| Nobs | 47 |  | 47 |  | 47 |  | 47 |  | 47 |  | 47 |  | 47 |  | 47 |  | 47 |  | 47 |  |
| DF | 42 |  | 42 |  | 44 |  | 44 |  | 44 |  | 44 |  | 42 |  | 42 |  | 43 |  | 43 |  |
|  |  |  |  |  |  |  |  |  |  |  |  |  |  |  |  |  |  |  |  |  |
| **ENSO Measure** | **ENSO_ Dom** | | | | | | | | | | | | | | | | | | | |
|  |  |  |  |  |  |  |  |  |  |  |  |  |  |  |  |  |  |  |  |  |
| Deviance | 154536.7 |  | 47.7 |  | 62449.3 |  | 47.8 |  | 30216.4 |  | 48.1 |  | 103620.8 |  | 50.1 |  | 103450.1 |  | 49.5 |  |
| Deviance/DF | 3679.45 |  | 1.14 |  | 1419.30 |  | 1.09 |  | 686.74 |  | 1.09 |  | 2467.16 |  | 1.19 |  | 2405.82 |  | 1.15 |  |
|  |  |  |  |  |  |  |  |  |  |  |  |  |  |  |  |  |  |  |  |  |
| Parameters | **PRM** | **S** | **NBRM** | **S** | **PRM** | **S** | **NBRM** | **S** | **PRM** | **S** | **NBRM** | **S** | **PRM** | **S** | **NBRM** | **S** | **PRM** | **S** | **NBRM** | **S** |
| Intercept | 8.3968 | *** | 8.2656 | *** | 8.1640 | *** | 8.2680 | *** | 8.8216 | *** | 8.8380 | *** | 7.3130 |  | 6.1348 | *** | 8.0763 | *** | 7.4794 | *** |
| Trend1 | 0.0820 | *** | 0.0872 | *** | 0.0490 | *** | 0.0455 | *** | -0.0143 | *** | -0.0150 | *** | 0.0853 |  | 0.1317 | *** | 0.0348 | *** | 0.0573 | *** |
| Trend2 | -0.1938 | *** | -0.2048 | *** | NA |  | NA |  | NA |  | NA |  | -0.2966 |  | -0.4543 | *** | -0.9183 | *** | -1.2751 | ** |
| Vextre | -1.1523 | *** | -1.2502 | *** | NA |  | NA |  | NA |  | NA |  | -0.8241 |  | -1.2988 | ** | NA |  | NA |  |
| ENSO | 0.1222 | *** | 0.0913 | * | 0.2022 | *** | 0.1771 | *** | 0.0699 | *** | 0.0616 | NS | -0.0139 |  | 0.0296 | NS | 0.0323 | *** | -0.0159 | NS |
| Dispersion  | 1.0000 |  | 0.0891 |  | 1.0000 |  | 0.1074 |  | 1.0000 |  | 0.1437 |  | 1.0000 |  | 0.4022 |  | 1.0000 | *** | 0.3173 |  |
| Test |  |  |  |  |  |  |  |  |  |  |  |  |  |  |  |  |  |  |  |  |
| W: BLT vs  [ BLT + ENSO] | 21247.9 | *** | 3.1 | * | 20274.8 |  | 9.1 | *** | 814.0 | *** | 1.0 | NS | 96.4 |  | 0.1 | NS | 271.7 |  | 0.0 | NS |
|  |  |  |  |  |  |  |  |  |  |  |  |  |  |  |  |  |  |  |  |  |
| Nobs: Number of observations available for the model;  DF: Degrees of Freedom;  Test: Wald Test, W ; High value or number of stars means reject in favor of the last model used in the test, [ ];  S: Significance: P-value ≤ 0.01: *** ; 0.01 < P-value ≤ 0.05 : ** ; 0.05 ≤ P-value < 0.10 : * ; P-value >0.1 (NS)  : All confidence intervals at 95% confidence of the dispersion parameter do not include zero inside their boundaries  NA: Not available | | | | | | | | | | | | | | | | | | | | |

Coefficients and their approximate statistical significances are shown for both Poisson (PRM) and Negative Binomial Regression models (NBRM), using ENSO_Dom to represent the annual ENSO state.
